# Supplementary material for: IMPLEmenting a clinical practice guideline for acute low back pain evidence-based manageMENT in general practice (IMPLEMENT): Cluster randomised controlled trial study protocol
Source: Implement Sci. 2008 Feb 22;3:11. doi: 10.1186/1748-5908-3-11 (PMC2291069; doi:10.1186/1748-5908-3-11)
Supplement: Additional file 1 — Completed CONSORT checklist for the IMPLEMENT CRCT study protocol. The completed checklist of items from the CONSORT statement for cluster randomised trials for the sections: title and abstract, introduction, and methods. [file 1748-5908-3-11-S1.pdf]

CONSORT Checklist of items to include when reporting a cluster randomised trial

(Campbell MK, Elbourne DR, Altman DG: CONSORT statement: extension to cluster randomised trials. BMJ 2004, 328(7441):702-708.)

Paper section and topic	Item	Descriptor [section title][1]
**Title and abstract**		
Design	1	How participants were allocated to interventions (eg random allocation, randomised, or randomly assigned), specifying that allocation was based on clusters **[Title; Abstract]**.
**Introduction**		
Background	2	Scientific background and explanation of rationale **[Background]**, including the rationale for using a cluster design **[Trial design]**.
**Methods**		
Participants	3	Eligibility criteria for participants and clusters **[Inclusion criteria; Exclusion criteria]** and the settings and locations where the data were collected **[Recruitment of general practices; Recruitment of patient participants]**.
Interventions	4	Precise details of the interventions intended for each group, whether they pertain to the individual level, the cluster level, or both, and how **[Control group; Intervention group]** and when they were actually administered **[Timing of recruitment, intervention delivery and follow-up]**.
Objectives	5	Specific objectives and hypotheses and whether they pertain to the individual level, the cluster level, or both **[Trial objectives]**.
Outcomes	6	Report clearly defined primary and secondary outcome measures, whether they pertain to the individual level, the cluster level, or both **[Primary outcome measures; Secondary outcome measures; Table 1]**, and, when applicable, any methods used to enhance the quality of measurements (eg multiple observations, training of assessors) **[Data quality assurance]**.
Sample size	7	How total sample size was determined (including method of calculation, number of clusters, cluster size, a coefficient of intracluster correlation (ICC or k), and an indication of its uncertainty) and, when applicable, explanation of any interim analyses and stopping rules **[Sample size]**.
Randomization		
Sequence generation	8	Method used to generate the random allocation sequence, including details of any restriction (eg blocking, stratification, matching) **[Randomisation and allocation concealment]**.
Allocation concealment	9	Method used to implement the random allocation sequence, specifying that allocation was based on clusters rather than individuals and clarifying whether the sequence was concealed until interventions were assigned **[Randomisation and allocation concealment]**.
Implementation	10	Who generated the allocation sequence **[Randomisation and allocation concealment]**, who enrolled participants **[Recruitment of general practices; Recruitment of patient participants; Applying the eligibility criteria]**, and who assigned participants to their groups **[Randomisation and allocation concealment]**.
Blinding (masking)	11	Whether participants, those administering the interventions, and those assessing the outcomes were blinded to group assignment **[Blinding]**. If done, how the success of blinding was evaluated.
Statistical methods	12	Statistical methods used to compare groups for primary outcome(s) indicating how clustering was taken into account; methods for additional analyses, such as subgroup analyses and adjusted analyses **[Analyses]**.

[1] The bold text in square brackets refers to the section title within the publication.
